# Dual-functional porous and cisplatin-loaded polymethylmethacrylate cement for reconstruction of load-bearing bone defect kills bone tumor cells

**DOI:** 10.1016/j.bioactmat.2021.12.023

**Published:** 2021-12-29

**Authors:** Zhule Wang, Liebert Parreiras Nogueira, Håvard Jostein Haugen, Ingrid CM. Van Der Geest, Patricia Caetano de Almeida Rodrigues, Dennis Janssen, Thom Bitter, Jeroen J.J.P. van den Beucken, Sander CG. Leeuwenburgh

**Affiliations:** a Radboud University Medical Center, Department of Dentistry - Regenerative Biomaterials, Radboud Institute for Molecular Life Sciences, Nijmegen, the Netherlands; b University of Oslo, Department of Biomaterials, Institute of Clinical Dentistry, Faculty of Dentistry, Oslo, Norway; c Radboud University Medical Center, Department of Orthopedics, Radboud Institute for Health Sciences, Nijmegen, the Netherlands

**Keywords:** Porous polymethylmethacrylate cement, Bone tumor treatment, Local drug delivery, Chemotherapeutic drug, *Ex vivo* biomechanical assessment

## Abstract

Malignant bone tumors are usually treated by resection of tumor tissue followed by filling of the bone defect with bone graft substitutes. Polymethylmethacrylate (PMMA) cement is the most commonly used bone substitute in clinical orthopedics in view of its reliability. However, the dense nature of PMMA renders this biomaterial unsuitable for local delivery of chemotherapeutic drugs to limit the recurrence of bone tumors. Here, we introduce porosity into PMMA cement by adding carboxymethylcellulose (CMC) to facilitate such local delivery of chemotherapeutic drugs, while retaining sufficient mechanical properties for bone reconstruction in load-bearing sites. Our results show that the mechanical strength of PMMA-based cements gradually decreases with increasing CMC content. Upon incorporation of ≥3% CMC, the PMMA-based cements released up to 18% of the loaded cisplatin, in contrast to cements containing lower amounts of CMC which only released less than 2% of the cisplatin over 28 days. This release of cisplatin efficiently killed osteosarcoma cells *in vitro* and the fraction of dead cells increased to 91.3% at day 7, which confirms the retained chemotherapeutic activity of released cisplatin from these PMMA-based cements. Additionally, tibias filled with PMMA-based cements containing up to 3% of CMC exhibit comparable compressive strengths as compared to intact tibias. In conclusion, we demonstrate that PMMA cements can be rendered therapeutically active by introducing porosity using CMC to allow for release of cisplatin without compromising mechanical properties beyond critical levels. As such, these data suggest that our dual-functional PMMA-based cements represent a viable treatment option for filling bone defects after bone tumor resection in load-bearing sites.

## Highlights

• Dual-functional porous PMMA cements are developed by introducing CMC as both pore generator and drug vehicle for cisplatin.
• PMMA-based cements containing ≥3% CMC release sufficient amounts of chemotherapeutically active cisplatin.
• PMMA-based cements containing ≤3% CMC retain sufficient mechanical properties for bone reconstruction at load-bearing sites.

## Graphical abstract

**Figure:**
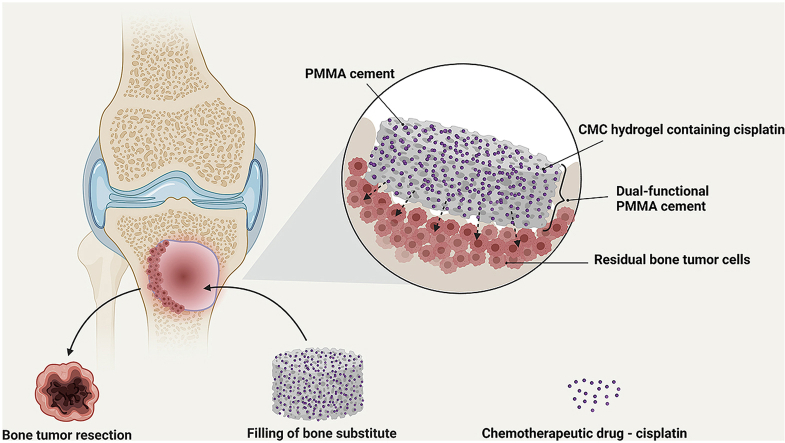

## 1 Introduction

Bone tumors represent one of the few fatal pathologies in orthopedics [1,2]. Primary bone tumors are less prevalent than more common cancer types such as lung, prostate, and breast cancer. However, these primary malignant bone tumors are quite lethal with low overall 5-year survival rates of less than 20% without adjuvant chemotherapy [3], and occur mainly in young people [4]. Owing to the emergence of chemotherapy and advanced surgical techniques for tumor resection, the overall 5-year survival rate of primary bone malignancies has meanwhile increased to 53–65% [5,6]. Primary malignant bone tumors first establish in bone or its accessory tissue and invade the surrounding tissue. Osteosarcoma, chondrosarcoma, fibrosarcoma, and Ewing's sarcoma are the most common primary malignant bone tumors, and their high lethality due to local recurrence remains a serious problem [7]. Despite the fact that limb salvage surgery and adjuvant chemotherapy have been widely undertaken for malignant bone tumor treatment, local recurrence rates of primary malignant bone tumors are between 10 and 15% [8,9]. Consequently, novel and effective treatments preventing recurrence of primary malignant bone tumors after surgical resection are urgently needed.

Most primary malignant bone tumors are osteolytic, and patients with severe bone lesions can suffer from pathological fractures at load-bearing sites, such as the distal femur and proximal tibia [10,11]. Thus, the standard treatment for malignant bone tumors involves surgical resection of the cancerous bone and filling of the resulting bone defect with a bone substitute material. This surgical approach is often combined with adjuvant chemotherapeutic or radiation treatment to reduce the risk of recurrence [[12], [13], [14]]. However, systemic chemotherapy is associated with severe side effects in vital organs (i.e. kidney and liver) [15], while high-dose radiotherapy can induce local osteonecrosis leading to failure of bone reconstruction at load-bearing sites [16]. Therefore, the ideal bone substitute for application in load-bearing sites should not only provide sufficient mechanical strength for reconstruction, but also exhibit local chemotherapeutic efficacy.

Polymethylmethacrylate (PMMA) cement is by far the most successful polymer-based bone substitute for orthopedic treatment of defects caused by primary bone tumors in load-bearing sites [[17], [18], [19]]. PMMA has many advantages, such as low cost, easy handling properties, moldability, high mechanical strength and appropriate biocompatibility. Upon surgical resection of bone tumors, PMMA is frequently used to reconstruct the normal anatomical structure at load-bearing sites (e.g. vertebra, femur and tibia) [18,[20], [21], [22]]. Furthermore, PMMA can elicit a local antitumor effect via exothermic polymerization causing thermal necrosis of tumor cells [23,24]. Even though this exothermic polymerization reaction is strong enough (>60 °C) to kill tumor cells, this local cytotoxic effect is limited to ∼3 mm around the PMMA [25]. In addition, the exothermic heat can cause thermal necrosis of healthy bone cells as well [26], which can hamper the mechanical interlocking between bone and PMMA [27].

To strengthen the chemotherapeutic efficacy of PMMA cements, research efforts have focused on the incorporation of chemotherapeutic agents into PMMA, including methotrexate (MTX), cisplatin and doxorubicin (DOX) [[28], [29], [30]]. Generally, these chemotherapeutic drugs were mixed with the powder component of PMMA cement and subsequently added to the liquid component. However, a major drawback of any drug-loaded PMMA cement involves the very limited amount of drug that can be released from these dense cements, as exemplified for instance by Wasserlauf et al. who reported maximum cumulative cisplatin release amounts of 3.4% over 6 months [31]. In addition, only very small amounts of drug can be exposed at the surface of dense PMMA cements [29,32]. Therefore, we hypothesized that the introduction of porosity is a prerequisite to become able to render PMMA cements chemotherapeutically active. Herein, we aimed to develop a dual-functional and chemotherapeutically active PMMA-based cement for filling of bone defects after bone tumor resection. This cement should release sufficient amounts of cisplatin to kill residual bone tumor cells without compromising mechanical properties beyond critical levels. We introduced porosity within PMMA by adding up to 4% of carboxymethylcellulose (CMC) to improve control over both loading and delivery kinetics of cisplatin. We show that PMMA-based cements containing 3% of CMC i) release substantial amounts of cisplatin, ii) effectively kill osteosarcoma cells *in vitro*, and iii) exhibit compressive strengths similar to intact tibias *ex vivo*. Overall, these promising results confirm the suitability of cisplatin- and CMC-functionalized PMMA cements for reconstructive treatment of primary malignant bone tumors in load-bearing sites.

## 2 Materials and methods

### 2.1 Materials

The commercial orthopedic bone cement (Antibiotic Simplex®, Stryker, USA; PMMA) used in this study consisted of two sterile components. The colorless liquid component (MMA monomer) comprised 97.5% methyl methacrylate monomer and 2.5% N, N-dimethyl para toluidine. 1.5 mg extra hydroquinone was added to the liquid component to prevent premature polymerization which may occur under the influence of strong light or elevated temperatures. The solid component (PMMA polymer) consisted of 75 wt% methylmethacrylate-styrene copolymer with 1.7 wt% benzoyl peroxide, 15 wt% polymethylmethacrylate and 10 wt% barium sulphate. Food-grade carboxymethylcellulose (AkzoNobel, Akucell AF2205, Arnhem, The Netherlands; CMC) was used to create porosity in PMMA cements. Cisplatin powder (99%, AK Scientific Inc., USA) was used as anticancer drug.

### 2.2 Preparation of PMMA-based cement

Porous PMMA-based cement was prepared following the method developed by De Wijn et al. [33]. The PMMA matrix phase was prepared by mixing the liquid and solid components of PMMA according to the instructions of the manufacturer. Briefly, 1.025 g solid powder was added to 0.5 mL of the liquid phase and manually stirred for 30 s. Then, the CMC hydrogel phase was prepared by dissolving CMC powder in Milli-Q. This hydrogel phase was mixed with the PMMA phase to produce cement samples with CMC contents ranging from 0 to 4 wt/wt% (see Table 1 for composition of the various experimental groups) [34,35]. The two phases were mixed with a spatula for the recommended operating time (4 min) at room temperature until a homogeneous paste was obtained. Afterward, to obtain cylindrical specimens, the homogeneous paste was inserted into a silicone rubber mold of 6 mm in diameter and 12 mm in height for 24 h and stored in normal temperature after curing. Cylindrical specimens (n = 4) with 6 mm diameter × 12 mm height were used for mechanical measurements, porosity analysis and morphological observations.

**Table 1:** Composition of porous PMMA of the experimental groups.

Group	PMMA matrix		Pore generator		
	Polymer (g)	Monomer (g)	CMC (g)	Water (mL)	CMC %
PMMA	2.05	1	0	0	0
PMMA+1%CMC	1.025	0.5	0.015	1.51	1
PMMA+2%CMC	1.025	0.5	0.03	1.495	2
PMMA+3%CMC	1.025	0.5	0.045	1.48	3
PMMA+4%CMC	1.025	0.5	0.06	1.465	4

### 2.3 Mechanical studies

All compression tests were performed using a tensile bench (810 MiniBionix IIVR, MTS, Eden Prairie, USA) at a cross-head speed of 0.5 mm/min. According to the International Standard ISO5833 (Implants for surgery – acrylic resin cements, 2002), cylindrical PMMA specimens with 6 mm diameter × 12 mm height were subjected to compression tests to determine the compressive (offset) yield strength and elastic modulus. Stress-strain curves were obtained until fracture of the specimens. The (offset) yield strength (σ_y_) and elastic modulus (E) were calculated using the formulas:

(1) $$\sigma_{\text{y}}\;=\text{ F/A;}$$

and

(2) E = σ/ε;

where F represents the load corresponding to 0.2% plastic deformation, A represents the cross-sectional area of the specimen; E corresponds to the slope of the linear part of the stress-strain curve, σ represents the stress applied on specimen, and ε represents the strain in the linear elastic region.

For fracture toughness measurements, the homogeneous paste of PMMA-based cement was inserted into silicone rubber molds (12 × 3 × 3 mm) to obtain rectangular PMMA specimens. After polymerization of the PMMA, the specimens were carefully removed from the molds and allowed to set for 24 h at room temperature. After setting, a dental handpiece (Type 5193, KaVo Dental Excellence, Germany) with a sharp steel drill was used to reproducibly prepare a single-edge notch (0.5 mm in width and 1 mm in depth) in the center of each specimen. A three-point bending test was performed using a previously described method [36] to determine fracture toughness values. To this end, the specimens were placed in a three-point bending testing model and the compressive load was centrally applied until the specimens fractured (originating from the notch). The fracture toughness (K_IC_) was calculated using the formula:

(3) K_IC_ = (PL/bw^1.5^) × f(a/w);

and

(4) f(a/w) = 3/α × (a/w)^0.5^ × {1.99 – (a/w)(1-a/w) × [2.15–3.93 × (a/w)+2.7 × (a/w)^2^]};

where α = 2 × (1+2a/w)(1-a/w)^3/2^; K_IC_ = stress intensity factor; P = load at fracture; L = span distance between the supports; w = width of the specimen; b = thickness of the specimen; and a = notch depth. Four specimens (n = 4) in each group were measured and the values were presented as mean ± standard deviation.

### 2.4 Material characterization

Fourier transform infrared spectrometry (FTIR; Spectrum One, PerkinElmer, USA) was used to characterize the molecular structure of the material and various cements. PMMA polymer, MMA monomer, CMC powders and PMMA-based cements containing 0, 1 and 4% CMC were analyzed over a range of 450–4000 cm^−1^ during 4 scans at a resolution of 4 cm^−1^. The FTIR spectra were normalized with an attenuated total reflection (ATR) correction.

### 2.5 Measurement of setting time and maximum exothermic temperature

The setting time of PMMA-based cement was measured using a standardized Gillmore needle protocol according to the ASTM C266-89. The heavy-weighted needle (300 g in weight and 1 mm in diameter) was used to determine the final setting time. After the homogeneous paste of PMMA-based cement was inserted into the bronze mold, the setting time was quantified by measuring the timepoint when the heavy-weighted needle did not make visible indentations anymore in the cement surface. The exothermic temperature during the polymerization reaction was recorded using a thermometer with a metallic needle (925K, testo BV, Netherlands). Once the homogeneous paste was inserted into the mold, the needle of the thermometer was placed at the center of the specimen to measure the maximum temperature. Four specimens (n = 4) in each group were measured at room temperature and the values were represented as mean ± standard deviation.

### 2.6 Ex vivo biomechanical analysis

Compression strength values of tibias filled with PMMA-based cements were assessed using an *ex vivo* model developed on the basis of previous studies [[37], [38], [39]]. Based on the prevalence of malignant bone tumors, we selected the tibia as representative load-bearing bone. We only used similar-sized and cylindrical diaphyses (proximal 40 mm in length and 7.5 mm in diameter) to decrease the variability between different specimens [40]. In order to mimic large bone defects resulting from surgical resection of malignant bone tumors in clinics, segmental bone defects in tibias were selected for further *ex vivo* compression tests. Additionally, since the location and size of bone tumors vary considerably [41], standardized and reproducible bone defects were created by semi-resection with the same width in tibia diaphyses as initial parameters of the defect shapes and sizes. With this *ex vivo* biomechanical set-up, we aimed to create bone defects at highly loaded skeletal sites to evaluate the reconstructive and reinforcing capacity of PMMA-based cements.

In brief, twenty-eight tibias from chickens were locally obtained from a supermarket (ALDI, Nijmegen, Netherlands). All soft tissues including skin, muscle, tendon and fascia were carefully removed using scalpels (BB73, AESCULAP, Melsungen, Germany) and tissue forceps (BD535R, AESCULAP, Melsungen, Germany). After the tibias were harvested, the condyles were cut using an aluminum oxide wheel (D3392, Cornad, Clausthal zellerfeld, Germany) and the mid-regions of diaphyses were kept for the compression tests. Furthermore, the ends of the diaphyses were flattened using a rotating sanding station with constant water cooling (M1725, Howell International Inc., Colorado, USA).

An ultrasonic device (Piezosurgery®, Mectron, Carasco, Italy) with a 0.55 mm micro-saw (OT-7 tip, Piezosurgery®, Mectron, Carsaco, Italy) was used to create a semi-resection bone defect (5 mm length) in the middle of the diaphysis. Subsequently, the bone marrow in the defect region was discarded and the bone defect was filled with PMMA-based cements containing up to 4% CMC as described above. Tibias were wrapped in gauze prewetted with PBS for 24 h to allow for complete curing of the cements. During the entire operation, all procedures were gently performed under moist conditions to minimize detrimental effects of heat generated by the micro-saw on bones samples and prevent the creation of notches in the bone samples. All *ex vivo* compression tests were performed using a tensile bench at a cross-head speed of 0.5 mm/min. The ultimate strength (σ_u_) and elastic modulus (E) were calculated using the formulas:

(5) $$\sigma_{u}=F/A;$$

and

(6) E=σ/ε;

where F represents the maximum load applied to specimens during the entire testing procedure, A represents the cross-sectional area of the specimen; σ represents the stress applied on specimen, and ε represents the strain in the linear elastic region. Intact tibias served as positive control and tibias with empty defects served as negative control. Four specimens (n = 4) were measured per experimental group and the values were presented as mean ± standard deviation.

### 2.7 Microtomography imaging and porosity analysis

PMMA-based cements containing up to 4% CMC were scanned on a Skyscan 2211 system (Bruker, Kontich, Belgium) which is equipped with a CCD-camera 11 Mpixels. The acquisition parameters were set to 55 kV, 280 μA, rotation step of 0.47° over 360° and exposure time of 0.650 ms per projection. The reconstruction of the virtual slices was performed with NRecon (v. 1.7.5, Bruker, Kontich, Belgium) with a final voxel size of 5.0 μm. The porosity study was performed using CT Analyser (CTAn 1.20.3.0+, Bruker, Kontich, Belgium). The 3D images were rendered using Avizo® 9.0 (Thermo Fisher Scientific, Waltham, MA, USA). Total porosity is the fraction of total void space in the sample; open porosity is the fraction of network of the pores that communicate with the boundaries of the sample; closed porosity is the fraction of pores that are not connected with the exterior; accessible porosity is the fraction of open pore structure that is accessible for particular diameter of a virtual sphere; pore size distribution is the fraction of the frequency of different pore diameters in the sample. In this work, virtual spheres with diameter ranging from 0 to 240 μm were used. Quantification of accessible porosity was conducted according to the method reported in a previous study [42]. The total porosity of cisplatin-loaded and cisplatin-free PMMA-based cements containing up to 4% CMC was also measured and calculated based on a gravimetry:

(7) Total porosity = [1−(M_PMMA_/ρ_PMMA_)/V_sample_] × 100%;

where M_PMMA_ is the weight of PMMA proportion in the specimen, ρ_PMMA_ (1.23 g/cm^3^) is the density of PMMA, and V_sample_ is the volume of the specimen.

### 2.8 Surface morphology

High resolution field emission scanning electron microscopy (FESEM; SM3010, Tokyo, Japan) was performed to evaluate the morphology of PMMA specimens at an acceleration voltage of 3.0 kV. Carbon tape was used to wrap both sides of each cylindrical specimen and to stabilize it on the individual stub for SEM analysis. After monitoring the top surface of the specimens, the cylindrical specimens were cut into two pieces using a rotary microtome (SP1600, Leica Microsystems Nussloch GmbH, Germany) to observe the cross-sectional surface of the specimens as well. Three specimens (n = 3) were observed for each experimental group. Furthermore, Energy-Dispersive X-ray Spectroscopy (EDX) was performed at an acceleration voltage of 15.0 kV to detect the presence of platinum and barium in the PMMA specimens.

### 2.9 Loading and release kinetics of cisplatin

A stock solution of anticancer drug cisplatin was prepared by dissolving cisplatin at 2.0 mg/mL in Milli-Q water. CMC powders (1–4 wt%) were added to these cisplatin solutions to obtain the CMC hydrogel (see Table S1 for PMMA-based cements containing cisplatin). Then, the CMC hydrogel phase was added to the PMMA phase at a ratio of 1:1 (wt/wt%) and inserted into silicone rubber molds after the homogeneous paste was obtained. After curing at room temperature for 2 h, the CMC-functionalized PMMA-based cements were stored before use. 2-[4-(2-Hydroxyethyl)-1-piperazinyl] ethanesulfonic acid (HEPES) buffer was used as immersion buffer (10 mM in Milli-Q water), the pH of which was adjusted to 6.5 to mimic the acidic cancer microenvironment [43,44]. The cured PMMA specimens were soaked in glass vials (PerkinElmer, USA) with 10 mL of HEPES buffer and placed in a bascule bath at 37 °C shaking at 90 rpm. After scheduled time points (1h, 1, 4, 7, 14 and 28 days), the supernatant was collected to determine platinum (Pt) concentrations and replaced with fresh HEPES buffer. The released Pt concentrations were quantified by inductively coupled plasma-mass-spectrometry (ICP-MS; UN2031, Panreac AppliChem, USA) after the supernatants were treated with 2 wt% nitric acid (HNO_3_). Cumulative Pt release percentage was calculated relative to the total amount of Pt added to each PMMA specimen. Four specimens (n = 4) in each group were measured and the values were presented as mean ± standard deviation.

### 2.10 In vitro anticancer activity of released Pt species

After collection of the releasates at day 1, the chemotherapeutic activity of the released Pt species was determined by indirect *in vitro* cell cultures with human osteosarcoma cells (MG-63, ATCC, Manassas, USA). MG-63 cells were cultured using Alpha (α)-MEM medium (A22571, Gibco) supplemented with 10% (v/v) fetal bovine serum (FBS, Lonza, Basel, Switzerland) and 1% penicillin and streptomycin (G418) in an incubator at 37 °C with 5% CO_2_. Freshly dissolved cisplatin in HEPES buffer (concentrations: 0–25 mM) was used as positive control; cells cultured in regular culture medium served as negative control. The metabolic activity of MG-63 cells was quantified as a measure for cytotoxicity using the Cell Counting Kit-8 (CCK-8; Sigma, Missouri, USA) assay. In brief, 5 × 10^3^ cells per well were seeded in 96-well plates with 50 μL growth medium. Then, another 50 μL of fresh cisplatin solution, releasates, HEPES buffer or growth medium was added to the corresponding well in quadruplicate (n = 4). At scheduled time points (1, 4 and 7 days), the CCK-8 assay was performed following the recommendations of the manufacturer. The cell survival rate was calculated using the formula: Survival rate (%)= (A_sample_-A_blank_)/(A_control_-A_blank_) x 100%, A = absorbance. The values were presented as mean ± standard deviation (n = 4).

The live/dead viability/cytotoxicity kit (L3224, Invitrogen molecular probes, Eugene, USA) was used to assess cell viability after culturing cells in the presence of the releasates. At scheduled time points, the cells were washed gently with Dulbecco's phosphate-buffered saline (D-PBS), and 150 μL of the working solution (approximately 2 μM calcein AM and 4 μM EthD-1) was added directly to the cells seeded on chamber slides (PEZGS0816, Millicell® EZ SLIDE, Darmstradt, Germany). After incubation for 30–45 min, the slides were placed on the working stage to view the labeled cells with fluorescence microscopy (3512001683, Carl Zeiss, Gottingen, Germany). To quantify the chemotherapeutic effects of the releasates on cell viability, the stained cells from three randomly selected fluorescent fields were automatically counted using Image J software (1.46r, National Institutes of Health, USA).

### 2.11 Statistical analysis

All data are presented as mean ± standard deviation. Statistical analyses were performed using GraphPad Prism® software (version 6.0, GraphPad Software Inc, California, USA). A one-way analysis of variance (ANOVA) with a *post-hoc* Tukey-Kramer multiple comparisons test was used to statistically analyze the mechanical tests and porosity. For the drug release study and the cell cytotoxicity assay, two-way analysis of variance (ANOVA) with a *post-hoc* Dunnett multiple comparisons test was used. P-values were considered as significantly different at p＜0.05.

## 3 Results

### 3.1 Mechanical properties of PMMA-based cements

**Fig. 1:**
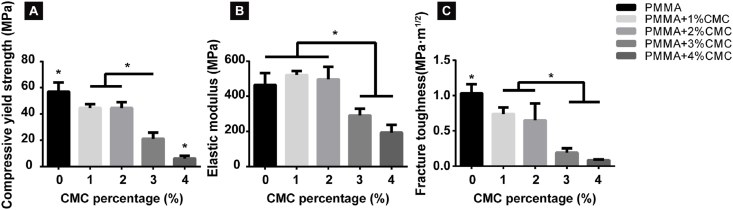
Mechanical properties of PMMA-based cements containing different amounts of CMC. (A) Compressive (offset) yield strength, (B) elastic modulus of PMMA-based cements containing up to 4% CMC content and (C) fracture toughness of PMMA-based cements containing up to 4% CMC content. An asterisk indicates statistically significant differences (*P < 0.05). Error bars represent standard deviations. At least four samples of each group (n ≥ 4) are used for all mechanical studies.

A representative stress-strain curve of PMMA cements is displayed in Fig. S1A. The compressive yield strength of the PMMA-based cements clearly decreased with increasing CMC content (Fig. 1A). As compared to CMC-free PMMA cement (57.0 ± 7.0 MPa), compressive yield strengths of CMC-containing PMMA cements were reduced upon incorporation of 1% CMC (44.5 ± 2.9 MPa; p < 0.05), 2% CMC (44.5 ± 4.5 MPa; p < 0.05), 3% CMC (21.1 ± 4.7 MPa; p < 0.05) and 4% CMC (6.1 ± 2.1 MPa; p < 0.05). The elastic moduli of the PMMA-based cements also strongly depended on the amount of CMC incorporation (Fig. 1B): while 1% and 2% CMC showed similar elastic moduli as CMC-free PMMA (range: 496.8 ± 71.0–521.4 ± 22.8 MPa; p > 0.05), the elastic moduli for PMMA-based cements with ≥3% CMC significantly decreased (range: 192.6 ± 44.7–290.6 ± 39.3 MPa; p < 0.05). Additionally, the incorporation of CMC decreased the fracture toughness (Fig. 1C), which ranged from 1.03 ± 0.12 MPa · m^1/2^ for CMC-free PMMA to 0.65 ± 0.24–0.74 ± 0.09 MPa · m^1/2^ for cements containing 1–2% CMC (p < 0.05), and 0.08 ± 0.01–0.2 ± 0.08 MPa · m^1/2^ for cements containing ≥3% CMC (p < 0.05). Loading of cisplatin into porous PMMA-based cements did not significantly affect the compressive yield strength of the resulting cements (Fig. S1B).

### 3.2 Characterization of materials

Figure S2 shows the spectra of PMMA polymer, MMA monomer, CMC and PMMA-based cements containing 0, 1 and 4% CMC. In the spectra of PMMA, absorptions corresponding to stretching vibration of –C=C- at 1645 cm^−1^ were detected in MMA monomer, and the absorption of stretching vibrations of C–O–C at 1150 cm^−1^ and CH_3_ at 740 and 1432 cm^−1^ were detected in PMMA after polymerization (Fig. S2A). In the CMC spectrum, the stretching vibrations of C–O was observed at 1590 cm^−1^. In the spectra of PMMA-based cements containing 1 and 4% CMC, absorptions at 1645 cm^−1^ corresponding to –C=C- bonds in MMA monomer were not detected, while the characteristic group of CMC was observed at 1590 cm^−1^ (Fig. S2B).

### 3.3 Setting time and exothermic temperature

**Fig. 2:**
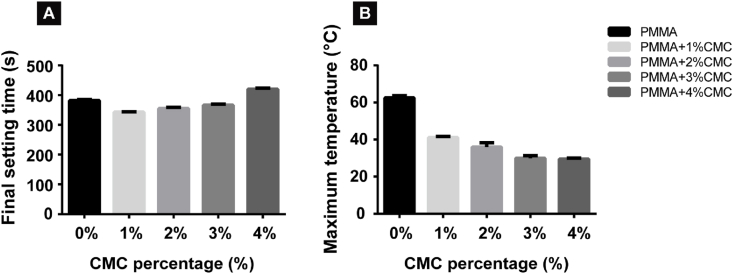
Setting time and maximum temperature during setting of PMMA-based cements containing 0–4% CMC. (A) Final setting time of PMMA-based cements and (B) maximum temperature at the center of the PMMA-based cement specimens. Error bars represent standard deviations. Four samples of each group (n = 4) are used for the quantitative analysis.

Final setting times of PMMA-based cements ranged from 342 ± 2.4 s to 419 ± 5.8 s for the various experimental groups (Fig. 2A). The setting time was slightly reduced by minor amounts of CMC (≤3%), whereas addition of 4% CMC resulted into a slight increase in setting time. The incorporation of CMC reduced the maximum temperature during setting of the PMMA cement from 62.5 ± 1.2 °C for CMC-free cement to temperatures below 37 °C upon addition of 4% CMC. (Fig. 2B).

### 3.4 Ex vivo biomechanical assessment

**Fig. 3:**
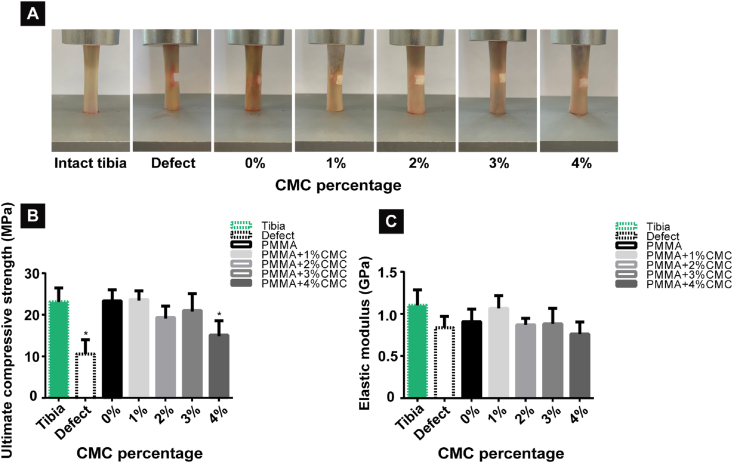
*Ex vivo* biomechanical evaluation of compressive strength of tibias filled with PMMA-based cements containing up to 4% CMC. (A) Representative photographs of compressive testing of tibias filled with PMMA-based cements including intact and defected tibias as controls, (B) ultimate compressive strength and (C) elastic modulus of different experimental groups. An asterisk indicates statistically significant differences (*P < 0.05). Error bars represent standard deviations. Four samples of each group (n = 4) are used for all quantitative studies.

Fig. 3A displays representative photographs of the tibia subjected to *ex vivo* compression tests. The tibias filled with PMMA-based cements containing 0–3% CMC showed similar ultimate compressive strengths (range: 19.3 ± 3.0–23.7 ± 2.3 MPa, p > 0.05) compared to intact tibia (23.3 ± 3.3 MPa, p > 0.05). However, the ultimate compressive strength of the negative control (tibia with empty defect) and tibias filled with PMMA-based cement containing 4% CMC were significantly reduced (range: 10.8 ± 3.4–15.1 ± 3.7 MPa, p < 0.05) (Fig. 3B). The elastic moduli of all tested experimental groups were statistically similar to intact tibias (1.10 ± 0.18 GPa, p > 0.05) and the negative control (0.84 ± 0.13 GPa, p > 0.05) (Fig. 3C).

### 3.5 Porosity of PMMA-based cements

**Fig. 4:**
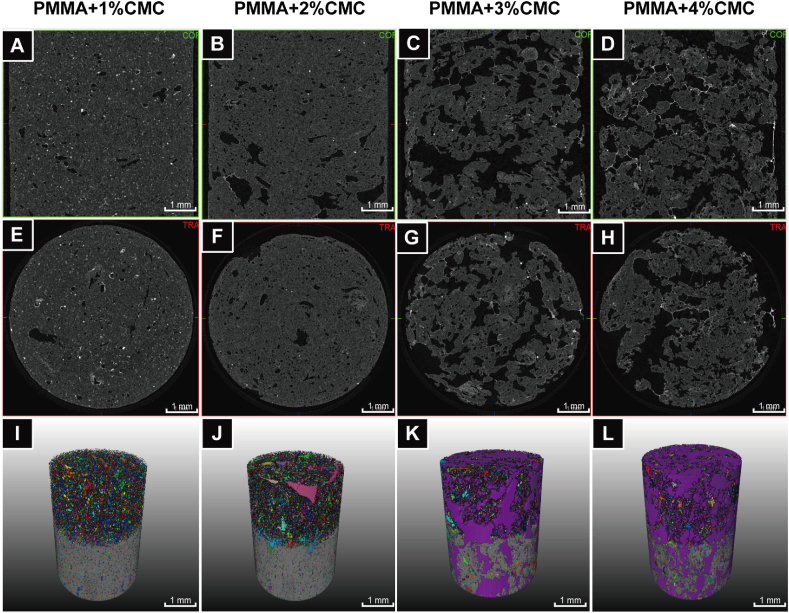
Representative micro-CT images and reconstructions of PMMA-based cements containing different amounts of CMC. (A–D) 2D micro-CT X-ray images of longitudinal sections, (E–H) 2D micro-CT X-ray images of transversal cross-sections and (I–L) 3D reconstructions of PMMA-based cements with different CMC content (1–4%). The colorful shapes above display the plenty of pores in the porous PMMA specimens. All specimens have a cross-sectional diameter of 6 mm. Scale bars correspond to 1 mm.

Fig. 4 presents representative micro-CT images of the porous PMMA-based cements as a function of CMC content (1–4%) for longitudinal (Fig. 4A–D) and transversal cross-sections (Fig. 4E–H). An apparent increase in porosity was observed with increasing CMC content, especially for PMMA-based cements with ≥3% CMC. For these PMMA-based cements, large pores with irregular dimensions and an interconnected structure were observed. Using 3D reconstructions, large numbers of small secluded pores were identified upon incorporation of 1–2% CMC, while 3–4% CMC incorporation showed large and interconnected pores (Fig. 4I-L and representative videos in Fig. S6).

**Fig. 5:**
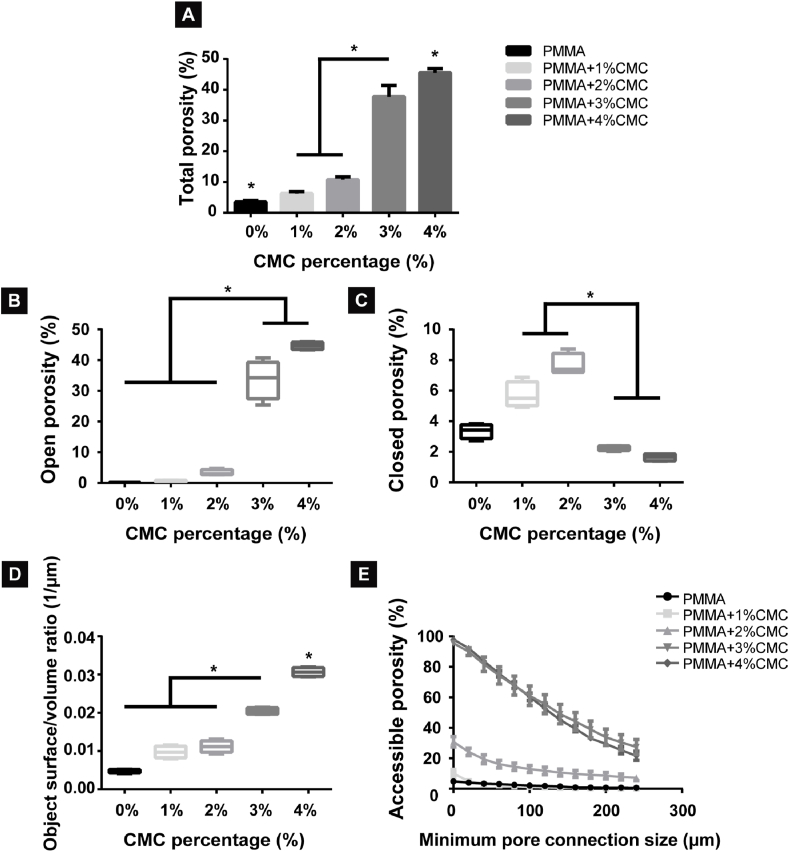
Quantitative analysis of porosity of PMMA-based cements with different amounts of CMC content. (A) Total porosity of PMMA-based cements, (B) percentage of open porosity, (C) percentage of closed porosity, (D) the ratio of object surface and volume and (E) percentage of accessible porosity of PMMA-based cements. (A–E) were analyzed and calculated by using micro-CT and Amira-Avizo 3D Software. An asterisk indicates statistically significant differences (*P < 0.05). Error bars represent standard deviations. Four samples of each group (n = 4) are used for all quantitative porosity studies.

The total porosity of PMMA-based cements increased from 3.5% for PMMA to 6.3–10.8% for cements containing 1–2% CMC (p < 0.05), 37.8% for 3% CMC (p < 0.05) and 45.6% for 4% CMC (Fig. 5A). The total porosity of cisplatin-loaded PMMA-based cements was statistically similar to PMMA-based cements without cisplatin (p > 0.05) (Fig. S3). The percentage and volume of the open porosity increased with increasing CMC content (Figs. 5B and S4A), leading to an amount of highly accessible porosity of 21.6–27.4% with a minimal pore connection size of 240 μm for PMMA-based cements containing ≥3% CMC (Fig. 5E). In contrast, the amount of closed porosity gradually increased from 3.4% for CMC-free cement to 7.7% for PMMA containing 2% CMC, but dropped to negligible amounts for CMC contents ≥3% (Figs. 5C and S4B). PMMA-based cements containing ≥3% CMC showed significantly higher object surface/volume ratios compared to other groups (p < 0.05) (Fig. 5D). The variations and standard deviations of the frequency of the pore size distributions were small within the same group, and the pore diameters of PMMA-based cements with up to 4% CMC mainly remained within 30–40 μm with frequencies of 36.9–39.9% for 30 μm, 19.4–21.8% for 35 μm and 9.6–13.1% for 40 μm (p > 0.05; Figs. S5A–E).

### 3.6 Morphological characterization of PMMA-based cements

**Fig. 6:**
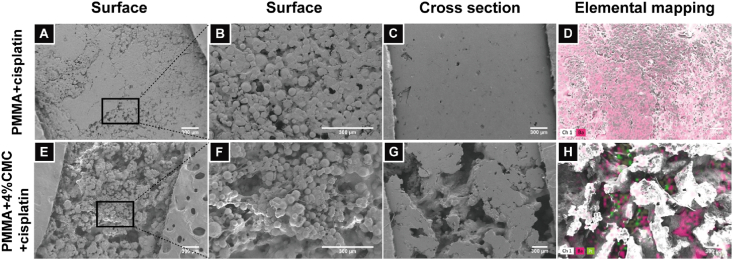
Surface morphologies of PMMA + cisplatin and PMMA+4%CMC + cisplatin. Representative scanning electron micrographs of the surface (A–B), cross sectional area (C) and elemental mapping images (D) of PMMA + cisplatin; Representative scanning electron micrographs of the surface (E–F), the cross sectional area (G) and elemental mapping images for barium (dark red) and platinum (green) (H) of PMMA+4%CMC + cisplatin. Scale bars correspond to 300 μm in all the micrographs. (For interpretation of the references to color in this figure legend, the reader is referred to the Web version of this article.)

Surface morphologies of the cisplatin-loaded PMMA-based cement containing 0% CMC (Fig. 6A–D) and 4% CMC (Fig. 6E–H) were characterized using scanning electron microscopy. Compared to the smooth and dense surface of CMC-free PMMA cement (Fig. 6A and B), the surface of the PMMA-based cement containing 4% CMC was more rough and porous (Fig. 6E and F). The transversal cross-sectional images also revealed an abundant presence of pore structures penetrating into the specimens for PMMA-based cement containing 4% CMC (Fig. 6G). The morphology of PMMA-based cement with 3% CMC were similar to 4% CMC group, while PMMA-based cements with 1–2% CMC and pure PMMA cement showed large morphological similarity. Using EDX for elemental analysis, barium (Ba) and platinum (Pt) were mapped on the surface of the cisplatin-loaded PMMA-based cements. The elemental distribution of Ba was homogeneous (Fig. 6D), whereas the Pt distribution was more heterogeneous, since Pt was mainly observed within the surface of pores of PMMA-based cement containing 4% CMC (Fig. 6H).

### 3.7 In vitro cisplatin release kinetics

**Fig. 7:**
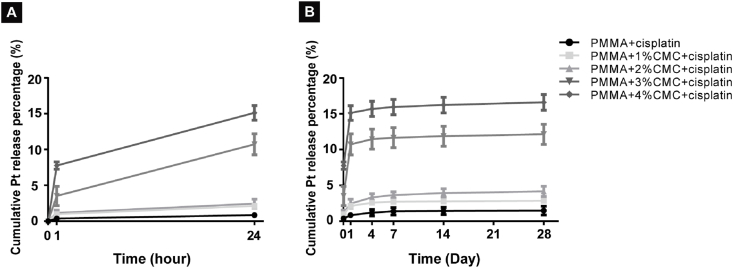
*In vitro* Pt release kinetics of cisplatin-loaded PMMA-based cements with different CMC content. Cumulative Pt release percentages of cisplatin-loaded PMMA-based cements containing up to 4% CMC in (A) the first 24 h, and (B) a period of 28 days. All the specimens were immersed in HEPES buffer (pH = 6.5). Error bars represent standard deviations. Four samples of each group (n = 4) are used for all quantitative studies.

The release of Pt from PMMA-based cements showed an initial burst within the first 24 h, followed by a phase of moderate but sustained release until 14 days. The amount of Pt bulk release within 24 h increased with increasing CMC content and ranged from 0.4 to 5.9 μg in total or 0.8–15.1% relative to the original amount of Pt (Figs. S7A and 7A). Subsequently, a sustained Pt release was observed from day 1 to day 14. The total cumulative Pt release over the entire study period of 28 days ranged from 1.8 ± 0.6% to 16.6 ± 1.1% (Fig. 7B) where the highest amounts of Pt release were observed for cisplatin-loaded PMMA-based cements containing 3% and 4% CMC (13.4% and 18%, respectively). Without CMC, PMMA-based cements hardly released any Pt over 28 days (total cumulative release of only 1.8%).

### 3.8 In vitro chemotherapeutic activity assay

**Fig. 8:**
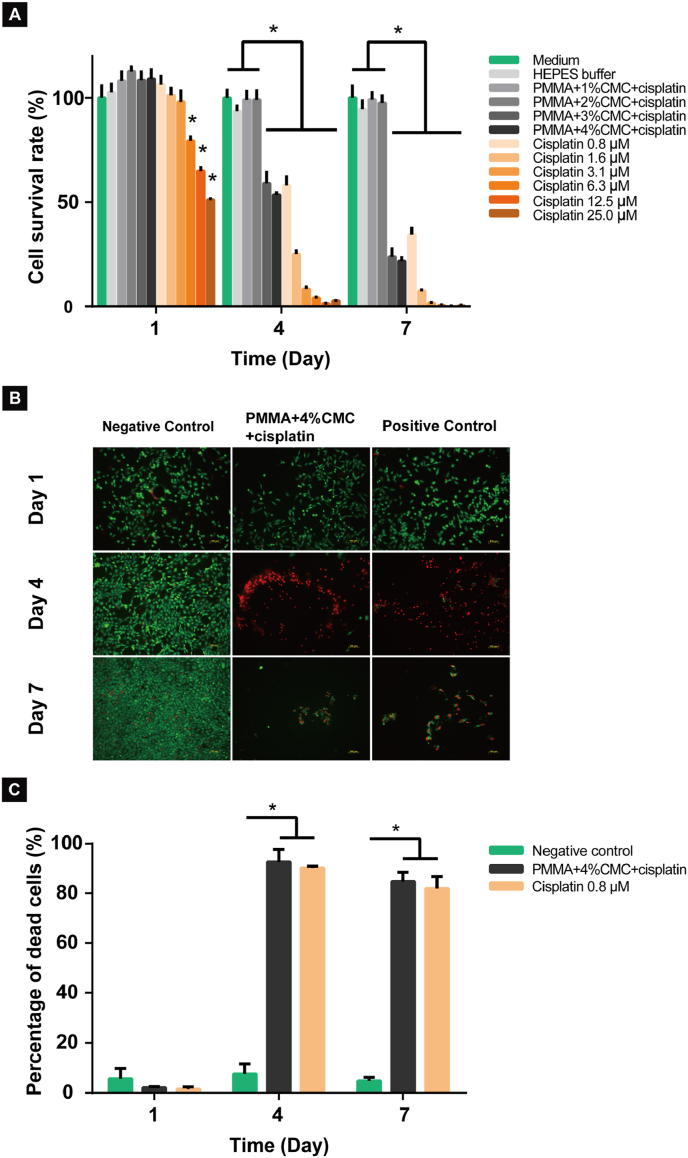
Effects of Pt release from PMMA-based cements with different CMC content (1–4%) on cultured MG-63 osteosarcoma cells. (A) Quantitative analysis of the cytotoxic effects of the released Pt species on cell proliferation determined by the CCK-8 assay, (B) representative fluorescence images of the cytotoxic effects of the releasates from cisplatin-loaded PMMA-based cement containing 4% CMC and free cisplatin (positive control: 0.8 μM) on cell viability assessed by live/dead staining and (C) quantitative analysis of the percentage of the dead cells based on live/dead staining. All experiments are performed at scheduled time points (1, 4 and 7 days). An asterisk indicates statistically significant differences (*P < 0.05). Error bars represent standard deviations. Four samples of each group (n = 4) are used for all quantitative studies. Scale bars correspond to 100 μm.

Exposure of MG-63 osteosarcoma cells to dissolved cisplatin and Pt releasates from PMMA-based cements after 1 day showed concentration-dependent effects on cell survival rates. At 1 day, no significant differences in cell survival rates were observed upon exposure to the releasates from PMMA-based cements containing 0–4% CMC (range: 108.2–112.7%, p > 0.05), positive control (dissolved cisplatin: 0.8–3.1 μM) (range: 98.1–106.4%, p > 0.05) and negative control (common culture medium) (range: 100–105.3%, p > 0.05), whereas dissolved cisplatin (6.3–25.0 μM) exhibited obvious cytotoxic effects on cell survival. At 4 and 7 days, significant chemotherapeutic effects on survival rates of osteosarcoma cells were observed for releasates from PMMA-based cements containing 3% and 4% CMC, which displayed a similar manner to freshly dissolved cisplatin (0.8–25.0 μM) (p < 0.05), and no cytotoxicity was found for releasates from PMMA-based cements containing up to 2% CMC (p > 0.05) (Fig. 8A). Additionally, the representative fluorescence images of live/dead staining confirmed the obvious cytotoxic effect of the releasates from the PMMA-based cement containing 4% CMC and positive control (0.8 μM) on human osteosarcoma cells (Fig. 8B). Quantitative analysis of the dead cell fraction showed a large increase to 92.2 ± 8.3% and 84.8 ± 6.5% for PMMA-based cement containing 4% CMC at 4 and 7 days, respectively (p < 0.05; Fig. 8C).

## 4 Discussion

Tumor recurrence is still the biggest challenge when treating malignant tumors, especially in case of primary bone tumors such as Ewing sarcoma characterized by recurrence rates of 30–40% [9,45]. Tumor cells remaining in the bone defect or surrounding tissue after bone tumor resection will considerably increase the risk of local tumor recurrence. In order to eliminate residual tumor cells and decrease side effects caused by systemic administration of chemotherapeutic drugs, bone substitute biomaterials such as PMMA bone cement should ideally not only facilitate mechanical reconstruction, but also locally deliver chemotherapeutic drugs [46]. To date, only few studies have focused on the development of such dual-functional bone substitutes [47,48]. As a novel approach, we herein introduce CMC as both (i) pore generator and (ii) carrier for a chemotherapeutic drug. Our findings show that (i) the CMC content not only determines the total amount and interconnectivity of porosity within the PMMA cement, but also allows for release of substantial amounts of cytostatic Pt species which effectively kill osteosarcoma cells; (ii) the CMC-functionalized PMMA-based cements maintain sufficient mechanical strength for bone reconstruction.

High polymerization temperatures during setting of PMMA cements are considered advantageous for treatment of bone tumors. During implantation, PMMA polymerization reaction can release sufficient heat to induce thermal necrosis of tumor cells [49]. However, this necrotic effect of the exothermic polymerization reaction of PMMA is unspecific and can also cause undesired thermal necrosis of healthy tissue [50,51]. Our results showed that the measured maximum temperatures (T_max_) were in the range of body temperature for PMMA-based cements containing ≥2% CMC. This finding may be explained by the fact that less polymerization heat was developed per unit volume after adding CMC hydrogel into the reaction system. In addition, the introduction of hydrated CMC and resulting porosity improved heat conduction, which decreased the polymerization temperature. These results corroborate earlier findings which already suggested that the incorporation of CMC hydrogel decreases the polymerization temperature [35,52]. Although FTIR analysis could not detect MMA monomers after polymerization, low contents of residual MMA monomers must still be present in our cured cements since monomer-to-polymer conversion is known to be lower at reduced polymerization temperatures [53]. The final setting times of PMMA-based cements containing 1–3% CMC were reduced, whereas cements containing 4% CMC increased as compared to CMC-free cements. However, the final setting times of cements were hardly affected by the introduction of CMC, and cements containing up to 4% CMC exhibit setting times which are well acceptable for clinical use by allowing sufficient time for surgeons to manipulate the cement during surgical procedures.

As expected, the addition of CMC to PMMA bone cements decreased their mechanical properties due to the creation of porosity. Mechanical properties are vital for application in highly loaded skeletal sites. Malignant bone tumors frequently occur in the epiphysis and metaphysis of long bones, which are mainly composed of trabecular bone (∼80%) [54]. For this reason, not only the mechanical properties but also the structure of the bone filling material should be similar to trabecular bone. The compressive yield strength and elastic modulus of human cortical and trabecular bone are reported to be 100–130 MPa and 17.9–18.2 GPa, and 4–12 MPa and 50–800 MPa, respectively [55,56]. Conventional PMMA is a dense material, and has a compressive yield strength of approximately 120 MPa, which is almost similar to human cortical bone. In this study, we used CMC to create porosity for drug delivery purposes. As the aqueous hydrophilic CMC hydrogel was heterogeneously dispersed throughout the hydrophobic PMMA with adequate mixing, the CMC hydrogel formed a heterogeneous phase within the composite [57]. After drying of the aqueous CMC hydrogel, voids were left which formed pores [58] (Fig. 4). The introduction of these pores expectedly decreased the compressive mechanical strength [59]. Consistent with the porosity analysis (Fig. 5), compressive strengths of PMMA-based cements decreased with increasing CMC incorporation. Generally, mechanical properties of PMMA-based cements were comparable to values reported for human trabecular bone.

To investigate if the reduced compressive strength of CMC-functionalized PMMA cement compromised its bone reconstructive capacity, we further developed an *ex vivo* bone defect model. Using this *ex vivo* biomechanical test set-up, we showed that intact tibias and tibias filled with PMMA-based cement containing up to 3% CMC exhibited comparable ultimate compressive strength, and all experimental groups displayed similar stiffness. These results indicated that PMMA-based cements containing ≤3% CMC enabled successful reconstruction of bone defects in tibias despite their decreased compressive strengths, suggesting that the cements retained sufficient mechanical strength to facilitate bone reconstructive procedures in segmental defects. Moreover, we found that the elastic modulus also decreased upon incorporation of CMC. The stiffness of porous CMC-functionalized PMMA-based cements was similar to the stiffness of trabecular bone (50–800 MPa). In contrast to highly stiff CMC-free PMMA cement, the porous structure and reduced stiffness of CMC-functionalized PMMA-based cement shows a higher structural and mechanical similarity to trabecular bone, which might reduce the extent of stress shielding and corresponding failures at the bone-cement interface [56,60]. In addition, it is generally accepted that with the creation of open and interconnected porosity into PMMA [58,61], more vascular and bone ingrowth will occur compared to common PMMA, which leads to stronger mechanical interlocking and less fibrous layer formation [62].

The release of cisplatin was considerably increased by introducing porosity into PMMA-based cements using CMC. As hypothesized, we observed a strong correlation between the CMC content and the amount of interconnected porosity. Incomplete water uptake was observed in PMMA cement containing 1% CMC during mixing, which not only led to reduced drug loading efficiency but also to reduced open porosity to release the drug. For PMMA cements containing higher than 1% CMC, the total porosity did not reach 50%, which is likely caused by the generation of heat upon exothermal polymerization leading to water evaporation during mixing [63]. With increasing CMC content, the polymerization temperature decreased, resulting in reduced water evaporation during the mixing, and higher porosity in PMMA-based cements. Numerous interconnective pore structures were observed both in the longitudinal and transverse section of the PMMA-based cements containing ≥3% CMC. Importantly, the interconnective pore structures created open access for drug loading and subsequent release. Cisplatin release from PMMA-based cements with ≥3% CMC was 9-fold higher as compared to CMC-free PMMA, and approximately 4 times higher than PMMA-based cements with 1% and 2% CMC. This finding confirms that the open porosity in CMC-functionalized PMMA-based cement improves their capacity to store and release chemotherapeutic drugs such as cisplatin.

Most importantly, we observed that our cisplatin-loaded PMMA cements released cytostatic Pt species which were able to effectively kill osteosarcoma cells *in vitro* when CMC contents were higher than 2%. This finding demonstrates that the released cisplatin maintained its chemotherapeutic efficacy after interaction with the carboxyl groups of CMC [64], and exceeded the minimally required therapeutic concentration of the drug [29]. Previous studies found that cisplatin (3.3%) was released less effectively from PMMA cement compared to chemotherapeutic drugs such as methotrexate (6%∼) and doxorubicin (∼11%) [31,65]. However, this shortcoming of PMMA cements was overcome in our study by introducing porosity into PMMA cement, resulting in cisplatin release percentages of more than 16%. This value exceeded not only i) previously reported values for cisplatin release from PMMA cement (3.3%–7.6%), but also release extents reported for other chemotherapeutic drugs (∼11%) [30,32,66,67]. Pt was mainly released within the first 24 h, which was in line with previous studies [28,68,69]. We consider this burst-type release pattern beneficial for the targeted application, since it allows to quickly reach the effective concentration to kill residual tumor cells directly after surgical application of the cement [13,30].

Nevertheless, it should be emphasized that about 80% of the total amount of loaded cisplatin remained entrapped in the cement. The incomplete release is related to the hydrophobic nature of PMMA. Compared to hydrophilic material, water cannot penetrate PMMA to increase the total drug release. In this study, CMC was used as pore generator to create open paths in dense PMMA to release cisplatin. The data showed that 34–45% open porosity was created in the PMMA-based cements containing 3–4% CMC, and allowed for more than 16% of the cumulative drug release. These results further support the idea that only the drug particles on the open surface of cement can be released [70]. Additionally, Mestiri et al. found that approximately 52% of cisplatin was released from PMMA cement *in vivo*, which was much higher than *in vitro* release at similar loading amounts [71]. They concluded that increased vascularity and perfusion around the cement was responsible for this enhanced *in vivo* release. The vascularized connective tissue may invade into the pores which are not accessible for water in the bone substitute to facilitate the drug release [62]. Further animal studies are required to be undertaken to investigate the drug release of the cisplatin-loaded PMMA-based cements *in vivo*.

The dual-functional PMMA-based cement consists of commercial PMMA cement, low amounts of CMC and commonly-used chemotherapeutic drug (cisplatin). Simplex® P PMMA is a commercially available bone cement mainly used for cementation of prosthesis and filling of bone defects resulting from bone tumor resection in vertebral bodies and long bones. CMC is a GRAS (Generally Recognized as Safe) ingredient and has been verified to be non-cytotoxic and biocompatible *in vivo* [72,73]. In addition, cisplatin has been approved by the Food and Drug Administration (FDA) for the systemic chemotherapeutic treatment of malignant bone tumors in clinics [74]. Therefore, our dual-functional PMMA-based cement is composed of biomaterials that are relatively simple from a regulatory perspective, while its manufacturing procedure is also straightforward. In this study, we found that the mechanical strengths of PMMA-based cements were sufficient for tibia reconstruction at CMC contents lower than 4% (≤3%), and the concentrations of released cisplatin were sufficient for killing of tumor cells at CMC contents higher than 2% (≥3%). Hence, PMMA-based cements containing 3% CMC are considered optimal for further preclinical studies. The combined reconstructive capacity and chemotherapeutic efficacy of this dual-functional PMMA-based cement renders this novel biomaterial suitable for further translational studies on treatment of malignant bone tumors [[75], [76], [77]].

## 5 Conclusion

In this study, we developed a dual-functional PMMA-based cement by introducing CMC as both pore generator and delivery vehicle for anticancer drug cisplatin. PMMA-based cements containing ≥3% CMC released sufficient amounts of chemotherapeutically active cisplatin which effectively kill osteosarcoma cells *in vitro*. Moreover, their mechanical strength is still sufficient to allow for reconstruction of bone defects in load-bearing sites. Consequently, this novel dual-functional cement combines two properties that are crucial for effective treatment of malignant bone tumors.

## Declaration of competing interest

The authors declare that they have no conflict of interest.

## CRediT authorship contribution statement

**Zhule Wang:** Conceptualization, Methodology, Formal analysis, Investigation, Data curation, Writing – original draft, Writing – review & editing. **Liebert Parreiras Nogueira:** Methodology, Software, Data curation, Writing – review & editing. **Håvard Jostein Haugen:** Software, Writing – review & editing. **Ingrid CM. Van Der Geest:** Conceptualization, Supervision. **Patricia Caetano de Almeida Rodrigues:** Conceptualization. **Dennis Janssen:** Conceptualization, Supervision. **Thom Bitter:** Conceptualization. **Jeroen J.J.P. van den Beucken:** Conceptualization, Methodology, Supervision, Writing – review & editing. **Sander CG. Leeuwenburgh:** Conceptualization, Methodology, Funding acquisition, Supervision, Project administration, Writing – review & editing.

## Data Availability

All relevant data supporting the findings of this study are stored in a digital and structured lab journal (LabGuru: https://radboudumc.labguru.com/knowledge/projects/6) or available upon request from the corresponding author.
