# Supplementary material for: Dual-functional porous and cisplatin-loaded polymethylmethacrylate cement for reconstruction of load-bearing bone defect kills bone tumor cells
Source: Bioact Mater. 2021 Dec 29;15:120–30. doi: 10.1016/j.bioactmat.2021.12.023 (PMC8941180; doi:10.1016/j.bioactmat.2021.12.023)
Supplement: Multimedia component 3 [file mmc3.docx]

**Appendix A. Supplementary data**

Table S1 Composition of porous cisplatin-loaded PMMA-based cements of the experimental groups.


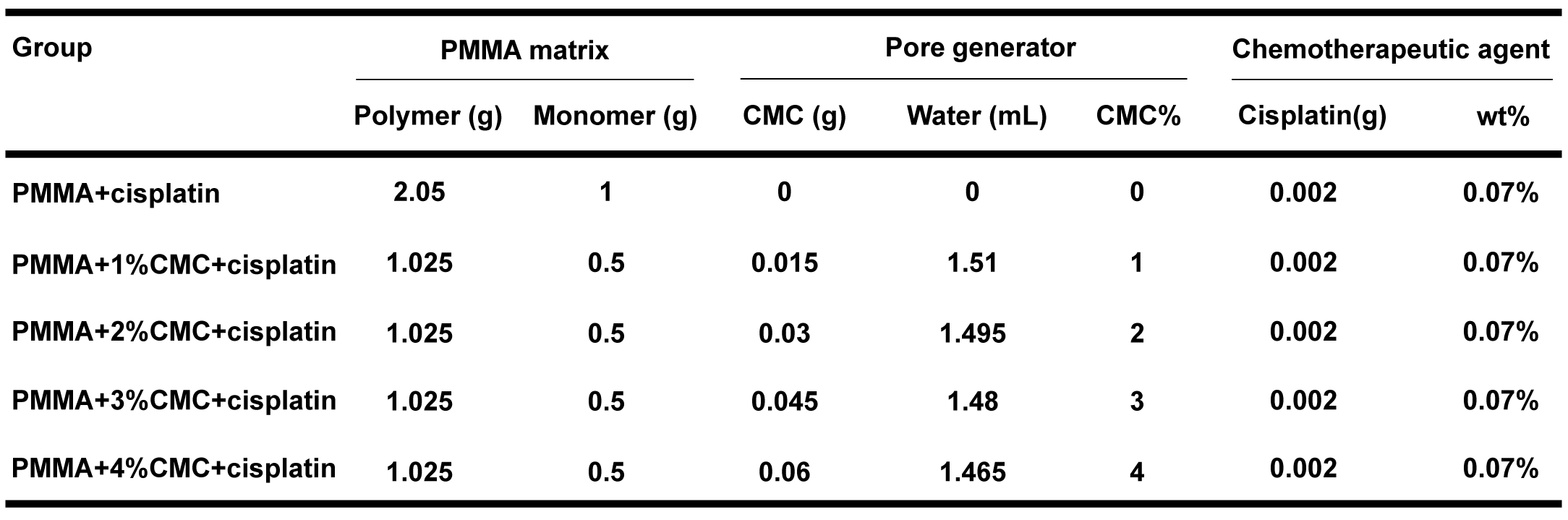


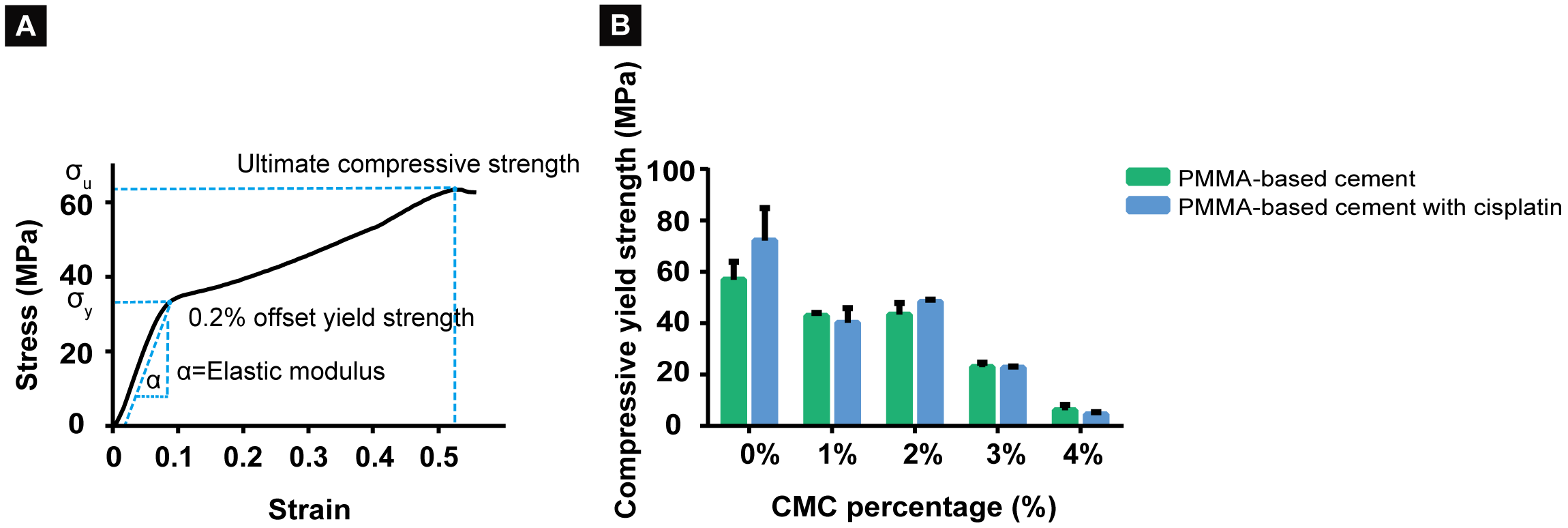


Figure S1 Compressive mechanical properties of PMMA-based cements with or without cisplatin. (A) Representative compressive stress-strain curve of polymer-based bone cements and the pattern used for calculation of yield strength and elastic modulus and (B) compressive (offset) yield strengths of PMMA-based cements containing up to 4% CMC. Error bars represent standard deviations. Four samples of each group (n =4) are used for the mechanical study.


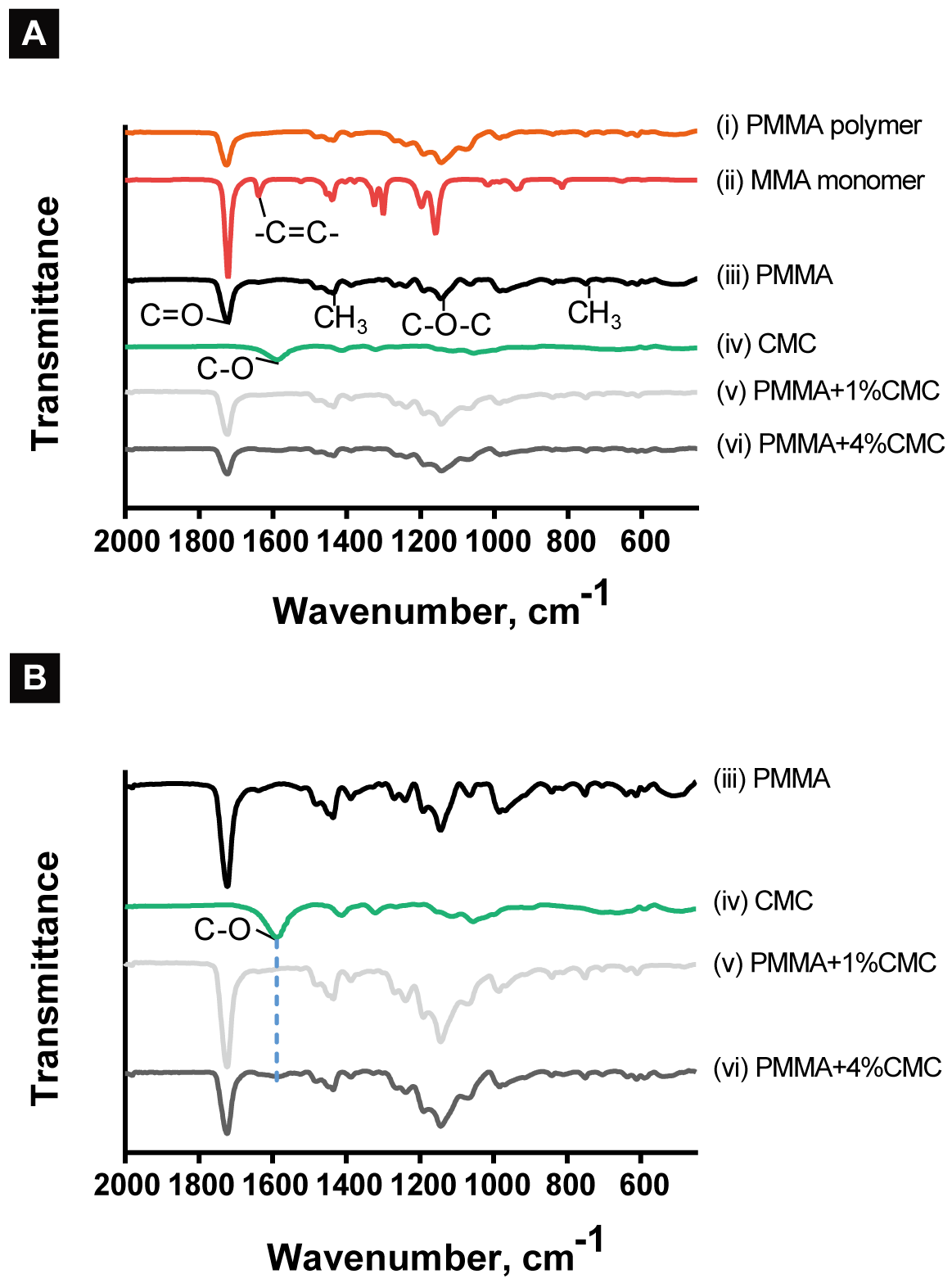


Figure S2 FTIR spectra for characteristic chemical groups in materials. (A) FTIR spectra of (i) PMMA polymer, (ii) MMA monomer, (iii) PMMA, (iv) CMC, (v) PMMA+1%CMC and (vi) PMMA+4%CMC. (B) Presence of characteristic chemical groups of CMC in the PMMA-based cements containing 1% and 4% CMC.


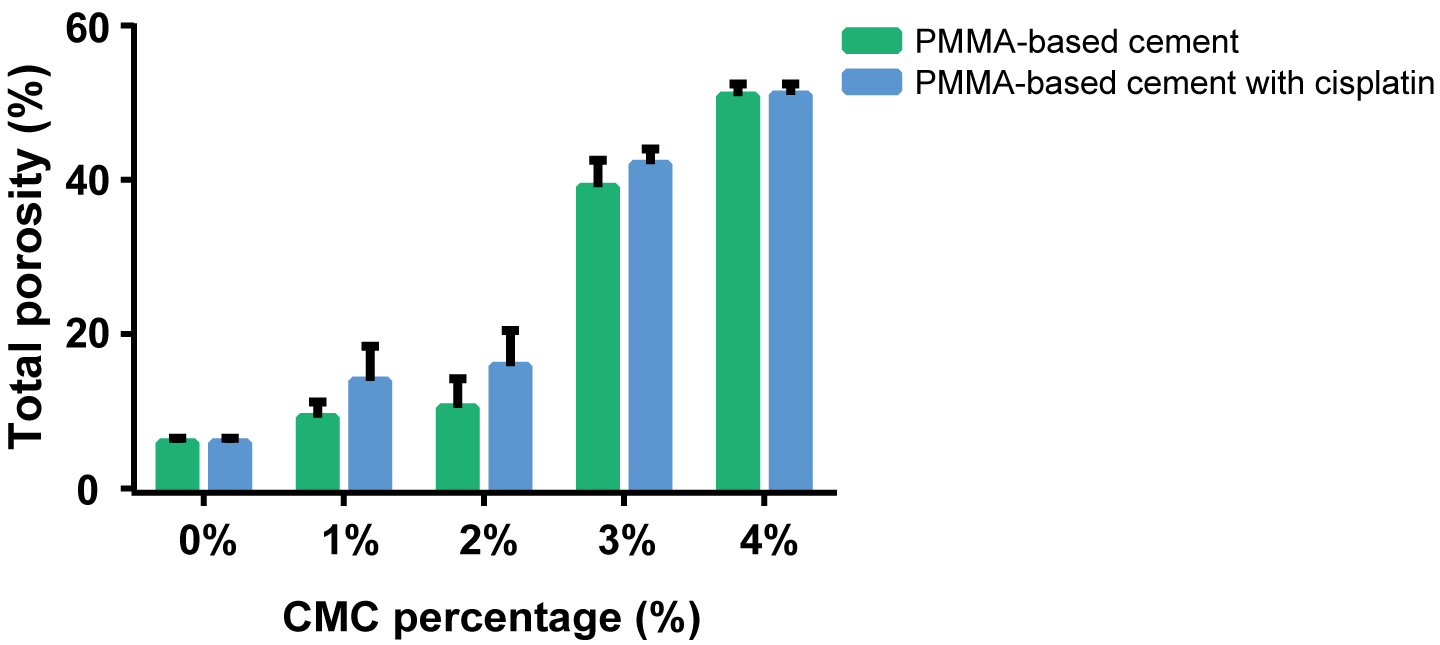


Figure S3 Quantitative comparison of total porosity between cisplatin-loaded and cisplatin-free PMMA-based cements containing up to 4% CMC calculated by using gravimetry. Error bars represent standard deviations. Four samples of each group (n ≥4) are used for the quantitative porosity study.


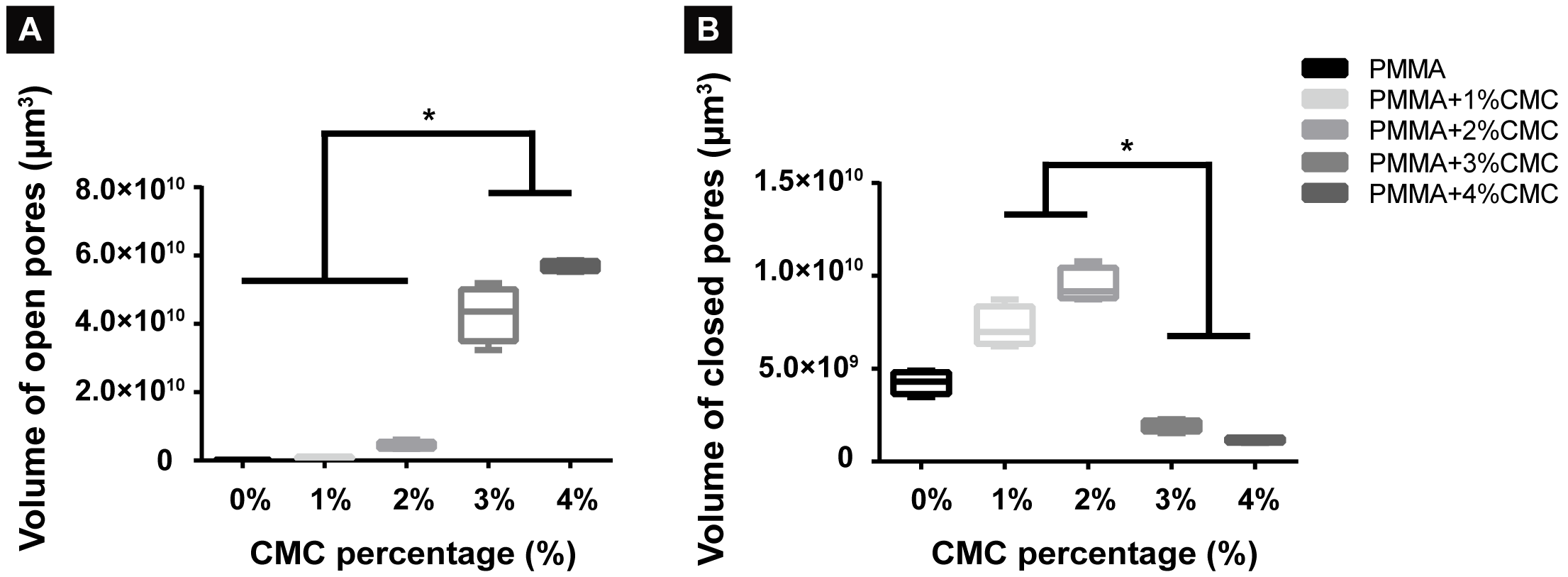


Figure S4 Quantitative analysis of porosity of PMMA-based cements with different amounts of CMC content. (A) volume of open pores and (B) volume of closed pores.


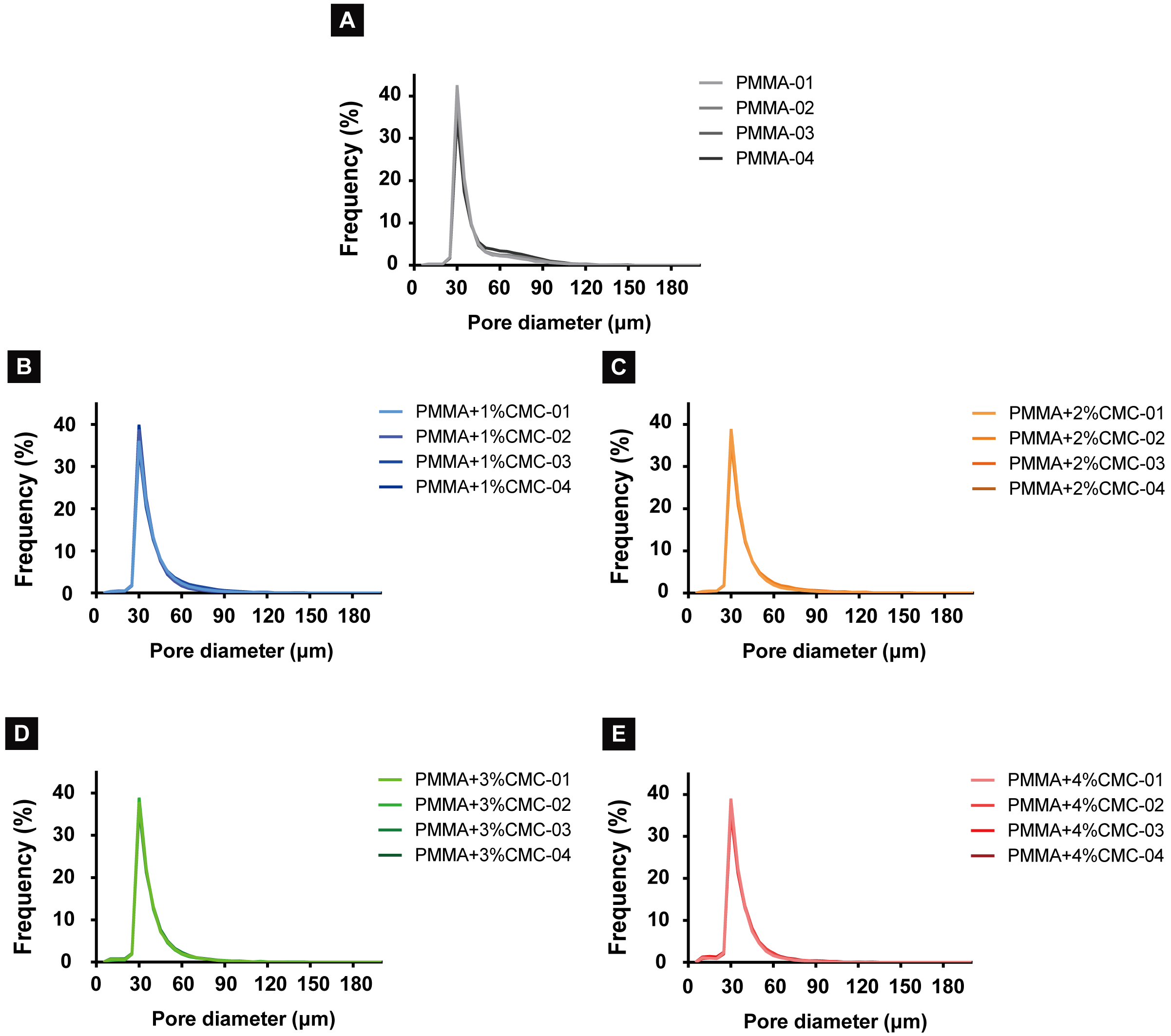
Figure S5 Quantitative analysis of the frequency of pore size distribution within PMMA-based cements containing up to 4% CMC. The frequency of pore size distributions in different specimens of PMMA-based cements containing (A) 0%, (B) 1%, (C) 2%, (D) 3% and (E) 4% CMC. Four samples of each group (n=4) are used for this quantitative study.

Figure S6 Representative videos of 3D reconstructions of CMC-free PMMA cement and PMMA-based cement containing 3% CMC.


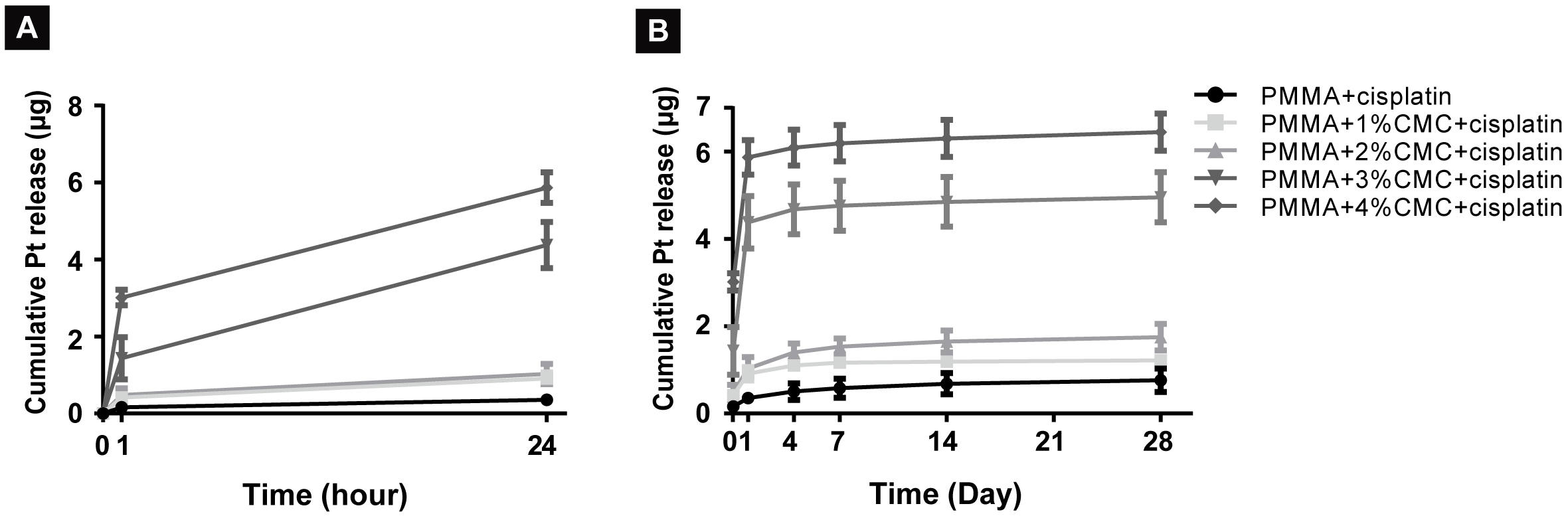


Figure S7 *In vitro* Pt release kinetics of cisplatin-loaded PMMA-based cements with different CMC content. Cumulative Pt release of cisplatin-loaded PMMA-based cements containing up to 4% CMC in (A) first 24 hours and (B) 28 days.
